# Breast cancer incidence and mortality in Tyrol/Austria after fifteen years of opportunistic mammography screening

**DOI:** 10.1186/1471-2458-10-86

**Published:** 2010-02-20

**Authors:** Willi Oberaigner, Wolfgang Buchberger, Thomas Frede, Rudolf Knapp, Christian Marth, Uwe Siebert

**Affiliations:** 1 Department of Clinical Epidemiology of the Tyrolean State Hospitals Ltd, Cancer Registry of Tyrol, Innsbruck, Austria; 2 Institute of Public Health, Medical Decision Making and Health Technology Assessment, Department of Public Health, Information Systems and Health Technology Assessment, UMIT - University for Health Sciences, Medical Informatics and Technology, Hall iT, Austria; 3 Oncotyrol - Center for Personalized Cancer Medicine, Innsbruck Medical University, Innsbruck, Austria; 4 University Hospital, Innsbruck, Austria; 5 Innsbruck Medical University, Department of Radiology, Innsbruck, Austria; 6 Kufstein County Hospital, Department of Radiology, Kufstein, Austria; 7 Innsbruck Medical University, Department of Obstetrics and Gynecology, Innsbruck, Austria; 8 Program in Health Decision Science, Department of Health Policy and Management, Harvard School of Public Health, Boston, MA, USA; 9 Institute for Technology Assessment and Department of Radiology, Massachusetts General Hospital, Harvard Medical School, Boston, MA, USA

## Abstract

**Background:**

The aim of this study was to analyse breast cancer incidence and mortality in Tyrol from 1970 to 2006, namely after performing more than a decade of opportunistic mammography screening and just before piloting an organised screening programme. Our investigation was conducted on a population level.

**Methods:**

To study time trends in breast cancer incidence and mortality, we applied the age-period-cohort model by Poisson regression to the official mortality data covering more than three decades from 1970 to 2006 and to the incidence data ranging from 1988 to 2006. In addition, for incidence data we analysed data on breast cancer staging and compared these with EU guidelines.

**Results:**

For the analysis of time trend in breast cancer mortality in age groups 40-79, an age-period-cohort model fits well and shows for years 2002-2006 a statistically significant reduction of 26% (95% CI 13%-36%) in breast cancer mortality as compared to 1992-1996.

We see only slight non-significant increases in breast cancer incidence. For the past five years, incidence data show a 10% proportion of in situ cases, and of 50% for cases in stages II+.

**Conclusions:**

The opportunistic breast cancer screening programme in Tyrol has only in part exploited the mortality reduction known for organised screening programmes. There seems to be potential for further improvement, and we recommend that an organised screening programme and a detailed screening database be introduced to collect all information needed to analyse the quality indicators suggested by the EU guidelines.

## Background

Breast cancer (BC) is the leading cause of female cancer death in all industrialised countries (and also worldwide) and the breast is also the leading incident cancer site for females [1]. Therefore, screening methods for BC are of greatest public health importance. Efficiency and efficacy of organised mammography screening programmes have been proven in large randomised trials conducted in Europe and North America. For several years already, organised mammography screening programmes have been recommended in the EU[2]. Austria is one of the European countries where up to 2006 no organised programmes were implemented, but where coverage in spontaneous mammography screening could have been rather high. In a micro-census conducted in Austria in 2006-2007, more than 80% of women aged 40-59 answered that they had had at least one mammography (ever) and more than 40% had had one in the past year[3]. However, it is known that self-reporting of screening usage overestimates true coverage [4], and first preliminary data from the organised mammography screening programme in Tyrol strongly confirm this interpretation. In 2006, the Austrian health minister declared mammography to be one of the top health agendas, and in July 2006 a decision was made to implement organised mammography screening programmes, in a first step in pilot regions, of which Tyrol is the largest.

In Tyrol, spontaneous mammography screening was set up around 1993. In July 1998 the "Working Group for Early Breast Cancer Detection for Tyrol" was established. Since that year recommendations including monthly breast self-examination, annual examination by a physician and annual mammography, if necessary with adjunctive breast sonogram, beginning at age 35-40, were formulated. Assessment is offered centrally by eight hospitals. Results of this strategy have been published [5]. Generally, in Austria spontaneous mammography screening is offered in the framework of general health exams done by general practitioners and of gynaecologic exams performed by gynaecologists in private practice. Women are referred for screening mammography mostly to radiologists in private practice. Both services are free of charge for women as of age 40. In 2007 and 2008, five pilot projects were launched in several of Austria's federal states in order to evaluate how to implement organised mammography screening. In Tyrol, a state screening programme was started in June 2007. In a one-year pilot phase, the methods were tested in two counties of Tyrol and in June 2008 the programme was extended to the whole state of Tyrol. The basic goal was to smoothly change the existing opportunistic screening system. Main characteristics are personal invitation, screening offered by radiologists and hospitals (out-patient departments), assessment at two hospitals, training of all partners and careful quality control by collecting all data in a central screening database and periodic inspection of data by a medical quality assurance group. No double reading was implemented.

We feel there is a need to publish the baseline characteristics of incidence and mortality in order to give a transparent public view of the situation in Tyrol before changing the mammography system. Although programme characteristics have not been collected to date, we can roughly judge the outcome achieved with the former spontaneous system by analysing time trends in incidence and mortality and by looking at stage shifts in BC cases. To our knowledge, it is not only in our country that spontaneous mammography screening is offered to women broadly, and there is ongoing discussion about whether the mammography system should be changed [6]. Therefore, it is of general interest to analyse the effects of spontaneous mammography screening offered free of charge to all women in a population. The analysis was only to be published now, because mortality and incidence data for female BC in Tyrol were published just a few months ago for the period to 2006 [7].

It was our aim to analyse BC incidence and mortality before changing the mammography system in Tyrol and to estimate the effects of the spontaneous programme offered free of charge to women for about fifteen years in order to have a public discussion of results before making the decision on whether and how to change the mammography system in Tyrol.

## Methods

### Mortality Data

Mortality data are collected by Statistics Austria for the whole of Austria [8]. In Austria, death certificates are issued by official, specially trained medical doctors, pathologists and forensic medical experts. Specialists at Statistics Austria, the federal institution for statistics in Austria, follow international guidelines and select one main diagnosis that led to death and assign it one ICD code (ICD9 up to 2001, ICD10 since 2002). All procedures concerning death certificates, data collection and coding are applied in a uniform way throughout Austria and are not state-specific. We analysed all female cases coded for cause of death BC as described above.

### Incidence Data

Incidence data have been collected by the Cancer Registry of Tyrol since year of diagnosis 1988 on a population-based perspective. Publication of incidence data in Cancer Incidence in Five Continents gives some hints for good completeness and validity of the database [9,10]. Registration is performed from a standardised questionnaire including sex, age, cancer site and histology, date of diagnosis, stage and basic information on primary treatment. Information on co-morbidity is not collected routinely. There are strict rules for collecting these variables in accordance with international guidelines. Either the questionnaire is completed by a physician or a Cancer Registry clerk collects data directly from clinical records in the treating hospital.

### Modelling of time trends

Time trends were analysed by fitting age-period-cohort (APC) models [11,12]. APC models allow separate effects to be estimated for age (A), period or year of death (P) and cohort (C) by means of Poisson regression. In a more formal sense we fit a series of models

where C=P-A, and ρ, denotes the mortality rate

The model is often written in antilogs as follows:

where α_A_' denotes the antilog of α_A _or α_A_' = exp(α_A_) etc.

As suggested by Clayton and Schifflers, a series of models is fit until adequate model fit is attained. We start with A alone and proceed by including P and/or C in the model if model fit is not sufficient without the extra term and inclusion of the term substantially improves goodness of fit. Goodness of fit is measured by deviance, which should be equal or close to the degrees of freedom (DF) if model fit is reasonably good.

For statistical analysis the number of BC deaths was aggregated in ten-year age groups and five-year period groups. We started with age group 40-49 and continued in ten-year age groups ending with age group 70-79, because we expected mammography screening to also affect this age group, bearing in mind that women aged 60-69 at the beginning of the screening programme around 1993 are now in age group 70-79. We have access to mortality data beginning in 1970. Our first period group was 1972-1976 in order to finish with the five year group 2002-2006. Our hypothesis was that the mortality rate decreased following the introduction of mammography screening around 1993. Thus, the reference category was the period 1992-1996. Consequently, because C = P-A, cohort groups begin with a cohort group centred at 1899.

To analyse the incidence time trend, we fitted an APC model for age groups 40 to 69, namely the age groups aimed at by the screening programme. The incidence data set begins with 1988. Therefore, we defined period groups 1988-1991 and then five-year period groups ending with 2002-2006.

For incidence data, we also analysed the proportion of in situ cases and the proportion of stages according to UICC and compared these with accepted levels given by EU guidelines [2].

Age-specific rates were calculated using official population numbers as denominators. Population data are also collected by Statistics Austria. Census data are available for the years 1971, 1981, 1991 and 2001; for intercensus years population figures are extrapolated based on birth, death and migration information. The female population of Tyrol in the census year 2001 was 345,757. The analysis was performed with Stata, Version 9; the APC model was set up using the procedure poisson for Poisson regression [13].

This study was conducted in conformity to the Helsinki Declaration [14].

## Results

**Figure 1:**
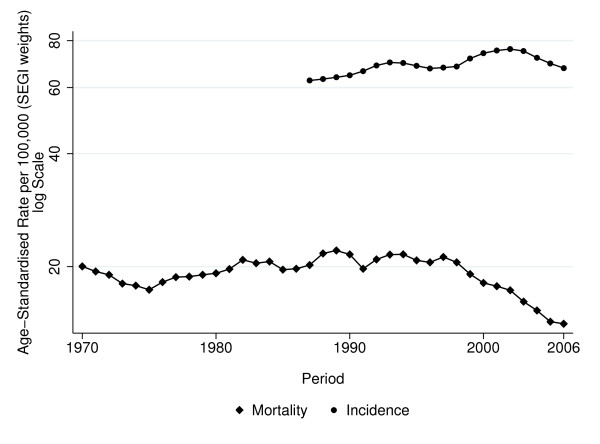
**Breast cancer mortality and incidence in Tyrol, age-standardised rates (SEGI weights) for all age groups**.

For an impression of overall BC mortality and incidence, Figure 1 shows the time trend in age-standardised mortality and incidence rates (for all age groups); age standardisation was based on world population proposed by SEGI and modified by Doll et al. [9]. The line of moving averages suggests a decline in mortality since 1998 and an increase in incidence until 2003, however on a purely descriptive level.

**Figure 2:**
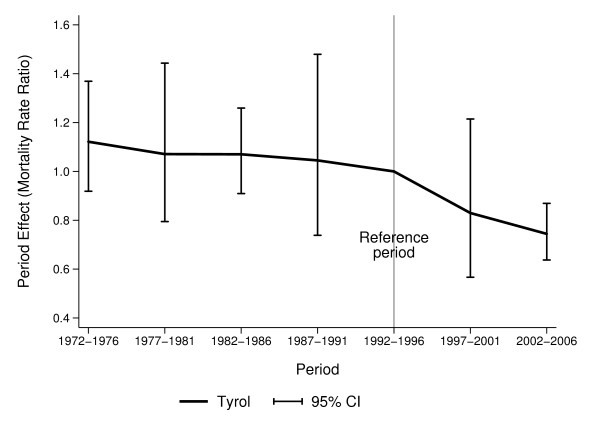
**Breast cancer mortality, APC model, P estimators; age groups 40-79**.

For a formal analysis of time trend, we fitted an APC model separately for mortality and incidence. For mortality, the final model includes terms for period and cohort because there are statistically significant cohort effects, model fit is very good (8 degrees of freedom, deviance 3.5). The resulting estimates for the APC model are described in Table 1 and Figure 2. Age effects, each compared to age group 40-49, are 2.15 for age group 50-59, 3.67 for age group 60-69 and 5.75 for age group 70-79. Period effects, each compared to 1992-1996, are about 1.05 before 1992, but 0.83 (95% CI 0.57, 1.21) for 1997-2001 and 0.74 (95% CI 0.64, 0.87) for 2002-2006. In general, the effects we report can be interpreted a change in mortality compared to the reference period, for example the effect of 0.83 for year 1997-2001 means a mortality reduction of 17% in 1997-2001 as compared to 1992-96. We also observe a strong cohort effect for cohorts born around 1920 and between 1930 and 1950 with relative risks at 1.4-1.8, each compared to the cohort centred at 1899.

**Table 1:** Model estimators for age, period and cohort given by the APC model, drift in period, for breast cancer mortality in Tyrol 1972-2006

	Estimator	95% CI	
**Age**			
40-49	1	Reference	
50-59	2.15	1.85	2.50
60-69	3.67	3.05	4.42
70-79	5.75	4.58	7.22
**Period**			
1972-1976	1.12	0.92	1.37
1977-1981	1.07	0.79	1.44
1982-1986	1.07	0.91	1.26
1987-1991	1.05	0.74	1.48
1992-1996	1	Reference	
1997-2001	0.83	0.57	1.21
2002-2006	0.74	0.64	0.87
**Cohort **(centred at)			
1899	1	reference	
1904	Collinearity		
1909	1.06	0.84	1.35
1914	1.20	0.92	1.56
1919	1.41	1.11	1.77
1924	1.32	0.98	1.77
1929	1.27	0.99	1.62
1934	1.60	1.13	2.28
1939	1.61	1.21	2.15
1944	1.80	1.18	2.74
1949	1.48	1.04	2.09
1954	1.55	0.90	2.66
1959	Drift		

* Because there is drift in period, there is no estimator for the last cohort centered at 1959

For incidence, the time period from 1988 to 2006 is much shorter. We modelled the time trend for age groups for which mammography screening was recommended, namely 40 to 69. The AP model reaches sufficient model fit with 8 degrees of freedom and a deviance of 8.3. Since adding a cohort parameter does not cause a significant improvement, we accepted the AP model. Period effects, each compared to 1992-1996, show a non-significant increase in BC incidence up to 1992, a slight but non-significant increase of 1.05 after 1996 and a steady situation during the last five years, see Table 2.

**Table 2:** Model estimators for age and period given by the AP model, for breast cancer incidence in Tyrol 1988-2006

	Estimator	95% CI	
**Age**			
40-49	1	Reference	
50-59	1.65	1.53	1.79
60-69	2.08	1.93	2.25
**Period**			
1988-1991	0.92	0.83	1.01
1992-1996	1	Reference	
1997-2001	1.05	0.96	1.14
2002-2006	1.05	0.96	1.14

Remark: There is no significant cohort effect. Therefore, the model was set up without cohort terms.

**Figure 3:**
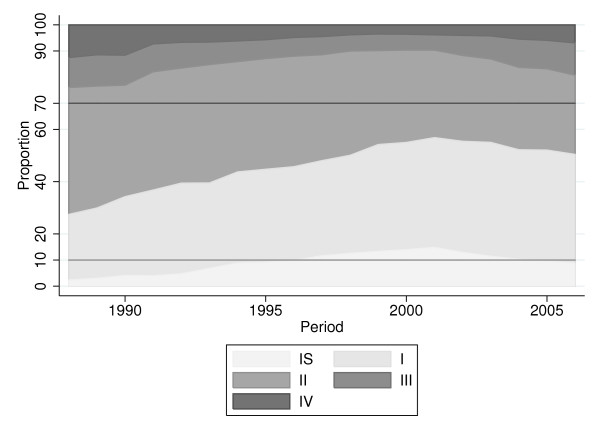
**Breast cancer incidence, proportion of cases by UICC staging, age groups 40-69**.

In addition, we also analysed some of the quality indicators proposed by EU guidelines [2]. The proportion of in situ cancers out of the total in situ and invasive cancers shows a steady increase from 5% around 1990 to 13% around 2000 and a slight decrease to 10% in recent years (see Figure 3). This time trend is consistent for all three age decades investigated (data not shown) and meets the 10% acceptable level given by EU guidelines. Figure 3 shows staging groups according to UICC. We see a clear stage shift towards early stages I and II up to around 2000 and a slight decrease afterwards. The EU acceptable proportion of stages II+ (30%) is clearly missed; in the last years the proportion of stages II+ in Tyrol was about 50%.

A more detailed analysis for the last five years shows a proportion of very small cancer with a size of less than 1 cm (TNM staging T1a,b) of 24% (age group 40-49), 22% (age group 50-59) and 19% (age group 60-69), the acceptable level according to EU guidelines being 25%. However, this information is not available for the 1990s.

The proportion of node-negative cases also increased to 58% (age group 40-49) and 53% (age group 50-69) at about 2000, the acceptable proportion in EU guidelines being 70%.

Applying a COX model to analyse the effect of adjuvant hormonal therapy resulted in a hazard ratio of 0.87 (95% CI 0.77, 0.99) adjusted for age and stage, this means that patients receiving adjuvant hormonal therapy have a 13% lower risk of death than patients without this therapy.

## Discussion

Our results indicate a significant decrease in BC mortality over the past five years, a nonsignificant slight increase in incidence and a stage shift towards early stages over the past fifteen years with some proportions in the range of the accepted levels given by EU guidelines.

### Strengths and Limitations

This observational study was conducted in the population of Tyrol. Mortality and incidence data were collected on a population level. Mortality data were provided by Statistics Austria. The quality of death certificates was very important for the conclusions drawn. In general, mortality statistics in Austria have been of high quality for decades [8]. Coding of cause of death is done according to international guidelines by specialists who attend international benchmarking exercises. As already stated above, death certificates are written by specially trained doctors.

Data on BC incidence on a population level are provided by the Cancer Registry of Tyrol, which is a member of IACR and whose data are published in Cancer Incidence in Five Continents, thus giving some evidence for good quality of incidence data [9,10,15]. Figures on completeness of incidence data show that for BC, in the past decade the proportion of death certificate-notified cases was 3.2% and the proportion of death certificate only cases 1.4% [7]. Both proportions allow the conclusion that completeness is good as compared to international data.

In addition, the proportion of cases with unknown or unspecified stage is less than 5% in age groups 40 to 69. When we analyse incidence data on a population level, we always encounter some cases that lack detailed information for various reasons. Since year of diagnosis 2004, the cancer registry includes a variable for mode of detection. However, the information is very incomplete, because in many cases we cannot obtain sufficient information from the hospital discharge records.

The model we fitted for analysis of the time trend shows very good model fit. This means we can trust the time trend parameters and can therefore draw reliable conclusions from the model. Moreover, the staging information used to describe stage shift should be reliable.

The main limitation is the lack of a screening database. Consequently, we do not have detailed information on screening performance parameters. Another weakness is that we have only some limited information on coverage from a micro-census performed in 2006/2007 and from a publication by Frede [5]. Both sources indicate a coverage of 70%. In Catalonia, Spain, Baré et al [16] reported also a coverage of 70% prior to introducing a screening programme and the authors investigated reasons for non-participation which can be very helpful in improving coverage. On the other side, it is known that self-reporting overestimates true coverage,[4] and a more realistic estimation could be a coverage of about 50%. This would fit to first preliminary data from the organised programme in Tyrol (data not shown). The lack of information on coverage and, of course, also on many other screening details was one of the reasons for changing the screening system, because we are convinced that a detailed knowledge of screening parameters is essential to draw valid conclusions in future. For staging distribution, the only source of information is the Cancer Registry dataset, whose focus was not to obtain information on screening indices but to concentrate on cancer cases.

### Time trend for mortality and incidence data, model fit

We applied an APC model that takes age, period and cohort effects into account and models time trends that differ from a linear trend. Such models are widely used in epidemiology, see for example [17,18]. Each of the models we applied for both mortality and incidence fits well on its own, and all parameters allowing judgment of model fit are reasonably good. Also, the graphs showing observed and predicted rates give additional evidence that the model describes the data very well and hence that we can rely on estimated parameters (graphs not shown). In summary, the time trends given by the models should adequately describe the situation we observe.

Concerning the decrease in BC incidence in recent years, Ravdin et al. [19] hypothesized for the USA that the reduction in hormone replacement therapy (HRT) is the main cause of the rapid decrease in BC incidence seen in the USA from 2003 to 2004. Also in Tyrol, we observe a decrease in BC incidence only in age groups 50+, although the decrease is not as sharp as in the USA. According to local experts, it is likely that also in Tyrol, part of the decrease in breast cancer incidence in the age group 50+ between 2004 and 2006 is due to a reduction in HRT.

The main question remains whether the significant 26% reduction in BC mortality over the past five years as compared to 1992-1996 is associated with opportunistic mammography screening. Both randomised trials and data from population-based organised mammography screening programmes provide clear evidence that organised mammography screening can reduce BC mortality. This was also communicated at an IARC international expert conference [20]. The extent of mortality reduction differs in detail, but in general is estimated to be between 20% and 25% [21-32]. However, for population-based organised programmes it is necessary to distinguish between various factors influencing BC mortality [33]. Some authors[33] estimate that a great part of BC mortality reduction (approximately 2/3 of reduction in England and Wales) is related to improvements in therapy, mainly the introduction of tamoxifen. For the USA, Berry et al. [34] found a range of 28% to 65% (median 46%) for the proportion of BC mortality reduction attributed to screening by modelling this proportion by seven independent investigators. For Tyrol, this would imply a mortality reduction of 7% to 17% attributable to screening. In addition, when comparing BC mortality trends between countries, stage distribution and differences in therapy also have to be discussed as factors influencing BC mortality at the population level.

Adjuvant therapy with tamoxifen was routinely introduced in Tyrol around 1985. We do not collect detailed information on BC therapy in the Cancer Registry, but we have an overall variable for adjuvant hormonal therapy. When we analysed the effect of adjuvant hormonal therapy in a COX model adjusted for age and stage, an overall effect of 13% was seen. With regard to time trend in survival rates, over the past fifteen years we observed an increase in relative five-year survival rates split by staging groups according to UICC (5% increase in stage I, 13% in stage II, and 5% in stage IV). Both observations are consistent with an estimated therapy effect on survival of between 10% and 15%, which is in line with the UK estimate [21]. Furthermore, as compared with EU guidelines, we miss some of the accepted levels (coverage, proportion of small cancers, proportion of II+ cancers, proportion of node-negative cancers). In conclusion, we estimate that less than half of the mortality reduction should be due to screening. This would mean that the screening effect is less than 13% and that, consequently, the opportunistic screening programme does not realise the potential of organised programmes, namely a mortality reduction of 20% - 25%.

However, when we compare BC data for Tyrol with quality indicators for mammography screening, we must remember that the BC data we analysed included all BC cases diagnosed in the population of Tyrol, not only those detected by opportunistic mammography screening. For example, Paci et al. [35] show a proportion of 53% for II+ breast cancer in the total population as opposed to 29% in the screen-detected subgroup.

Vutuc et al. recently analysed BC mortality in Austria [6]. The authors argue that BC screening is a plausible explanation for BC mortality reduction and doubt that a change in screening policy (meaning changing from opportunistic screening to an organised programme) would significantly improve the situation in Austria. We agree that BC screening is indeed one possible explanation for BC mortality reduction. However, if we take into consideration the fact that we have no detailed information on diagnostic performance or coverage for opportunistic BC screening in Austria, we feel it is absolutely necessary that detailed information on mammography screening be collected, at least for several years. We need to know all the well-established quality indices for BC screening[2] before we can draw a final conclusion on how to proceed with mammography screening in Austria.

Interestingly, the greatest reduction in BC mortality was observed in the age group 40 to 49. This differs somewhat from international data, where doubts still prevail on the efficacy of mammography screening in the age groups below 50, see for example [36,37]. Surrogate performance indicators like stage shift, cancer size less than 1 cm and proportion of node-negative cancer also showed a clear tendency towards better performance in the age group 40 to 49 as compared to the age groups 50 to 59 and 60 to 69. In addition, during the past decade, these indicators improved more quickly in the age group 40 to 49 (details not shown). One possible explanation is the wide-spread use of sonography as an adjunct to mammography in Tyrol. It has been shown by various authors that the additional use of sonography can improve cancer detection rates, especially in younger women and women with dense breasts. The relative percentage of carcinomas found in supplemental breast ultrasound examinations as a fraction of the total number of detected cancers was reported by four studies, with a mean percentage of 22.5% (15%-34%) [38].

In opportunistic screening in Tyrol, sonography was offered to women with dense breasts (ACR density grades 3 and 4) and with inconclusive findings on mammography [39]. In addition, women in the younger age groups are likely to go more frequently to their general practitioner or gynaecologist, which results in higher coverage by opportunistic screening [3].

The discussion in the USA after publishing the revised recommendation by U.S. Preventive Services Task Force [40,41] shows that it is very challenging and hard to understand by women to remove a service that was recommended for several years. Without well founded data, we feel it is not justified to stop screening in age class 40-49. We are collecting detailed data and will evaluate the balance between goods and harms during the next years.

Some of the EU recommendations like double reading and making an appointment for mammography when inviting women will not be part of the organised programme in Tyrol. Thus, further investigation will be needed to prove whether mammography screening has an effect on BC mortality, even in the absence of these EU recommendations.

## Conclusions

Up to now, in terms of BC mortality reduction our analysis shows that it is likely that the full potential of mammography screening has not yet been realised. In addition, available cancer registry data are not sufficient to assess the efficiency or efficacy of the current opportunistic screening programme. Therefore, to analyse surrogate indices like decrease in advanced stages and increase in early stages, interval cancer rates, and to investigate the cost efficiency of the established programme, it is absolutely necessary that a well-organised screening database be built up that contains all information needed to analyse the quality indicators suggested by the EU guidelines. In conclusion, we strongly advise that an organised mammography screening programme be introduced in Tyrol, namely one that will also allow a detailed analysis of the effects of mammography screening.

## List of Abbreviations

CI: confidence interval; AP: age-period model; APC: age-period-cohort model; BC: breast cancer; HRT: hormone replacement therapy.

## Acknowledgments

This work was supported by the ONCOTYROL Center for Personal Cancer Medicine. ONCOTYROL is a COMET Center funded by the Federal Ministry for Transport Innovation and Technology (BMVIT) and the Federal Ministry of Economics and Labour (BMWA), the Tyrolean Future Foundation (TZS) and the Land of Styria represented by the Styrian Business Promotion Agency (SFG) [and supported by UMIT - University for Health Sciences, Medical Informatics and Technology, Innsbruck Medical University, Tyrolean Health Insurance Fund and the Tyrolean Health Company.

## Competing interests

The authors declare that they have no competing interests.

## Authors' contributions

WO designed the study, performed the analysis and wrote the paper. US contributed to study design and writing of the paper. WB assisted in writing the paper, especially contributions on ultrasound and screening at age below 50. TF und RK contributed to the discussion part, especially from the radiology point of view. CM contributed to the methods, report and discussion part, especially from the gynaecological point of view. All authors reviewed and agreed to the final version of the manuscript.

## Pre-publication history

The pre-publication history for this paper can be accessed here:

http://www.biomedcentral.com/1471-2458/10/86/prepub
